# Developing Concept Maps as an Aid for Enhancing Analytical Thinking in Undergraduate Medical Students

**DOI:** 10.7759/cureus.92687

**Published:** 2025-09-19

**Authors:** Rati Tandon, Nahid Yasmin, Monica Baxla, Hare Krishna

**Affiliations:** 1 Anatomy, Mahatma Gandhi Medical College and Hospital, Jaipur, IND; 2 Anatomy, Santosh Medical College and Hospital, Ghaziabad, IND; 3 Anatomy, All India Institute of Medical Sciences, Jodhpur, IND

**Keywords:** analytical thinking, cognitive learning, concept maps, didactic lecture, meaningful learning

## Abstract

Introduction

Concept maps (CMs) are a sequential and graphical method of representing the learned material or topic by the learner. They are represented by the main headings contained in the boxes connected with arrows, and also contain linkers that keep joining the different connected topics. It helps in linking concepts and visualizing their relations. This might help in discovering new problem-solving and analytical thinking. The present study was conducted to sensitize students to using CMs as a learning tool and to assess their effect on learning.

Method

A cross-sectional prospective study was conducted on 150 first-year Bachelor of Medicine and Bachelor of Surgery (MBBS) students divided into two groups, I and II. Group I was taught only by didactic lecture, while group II was taught by both didactic lecture and concept map. Pre- and posttests were conducted for all groups with a prevalidated questionnaire. Feedback was obtained from both groups using a three-point Likert scale. Paired t-tests and Wilcoxon matched-pairs signed-rank tests were applied for statistical analysis according to the distribution of data. A p-value of <0.05 was considered statistically significant.

Result

Both groups had similar pretest scores, but the posttest scores showed a significant increase in scores of students in both groups I and II. We observed a significant improvement in knowledge (gain of marks) in group II, which used CMs, in comparison to group I, which was taught using only traditional didactic lectures. This improvement was more marked regarding high cognitive-type MCQs. We observed a significant increase in the performance of group II students (median = 7 (5.0-8.0)) in comparison to group I students (median = 3 (2.0-5.0)) (p-value < 0.0001). After analyzing the feedback questionnaire, we observed that 64.2% (96) and 55.2% (83) of the students agreed that the concept mapping exercise helped them to understand and correlate the topic more coherently, respectively.

Conclusion

Concept mapping is a better learning tool in comparison to the classical didactic lecture and demonstration method. It facilitates higher cognitive learning. It can be included in the medical undergraduate curriculum to facilitate meaningful learning.

## Introduction

Concept maps (CMs) are a sequential and graphical method of representing the learned material or topic by the student. They are represented by the main headings contained in the boxes connected with arrows and also contain linkers that keep on joining the different connected topics [1]. It helps in linking concepts and visualizing their relations. This might help in discovering new problem-solving and analytical thinking. CMs keep evolving as they become more detailed and keep on reconfiguring [2].

CMs are constructive tools in the study. There is evidence suggesting that CMs are a tool for assessing the knowledge of students using traditional methods and after using CMs in medical education. Concept mapping, as a learning and teaching strategy developed by Novak and Gowin (1984), is one strategy to promote meaningful learning. Novak’s work was based on the theories of Ausubel. Novak concluded that “meaningful learning involves the assimilation of new concepts and propositions into existing cognitive structures” [3]. The NMC's proposed medical curriculum for 2019 draws attention to self-directed learning, outcome-based learning, and lifelong learning of the student, along with skills required for proper patient care. CMs have been developed as a part of a new method of learning so that the various levels of domains of teaching and learning are covered. Understanding and implementing CMs as a learning technique takes time because they are frequently viewed as a novel approach to learning by both faculty and students.

This all will lead to better learning for students in basic sciences with integrated skills with clinical sciences [2]. It's time to take rote learning to meaningful learning. In rote learning, all the concepts need to be learned, while in meaningful learning, knowledge acquisition occurs in a way that the knowledge can be utilized in doing something [4]. CMs not only help in meaningful learning; rather, they have also become a part of curriculum development, course design, method of assessment, and teaching evaluation, as well as teaching applications [5]. Comparison of different CMs can be done in order to have a higher understanding of the topic [6]. The present study was conducted to sensitize students to use CMs as a learning tool and to assess their effect on learning. The objectives of the study were to compare the learning with CMs and traditional lecture-demonstration methods in the anatomy of the brachial plexus.

## Materials and methods

**Figure 1:**
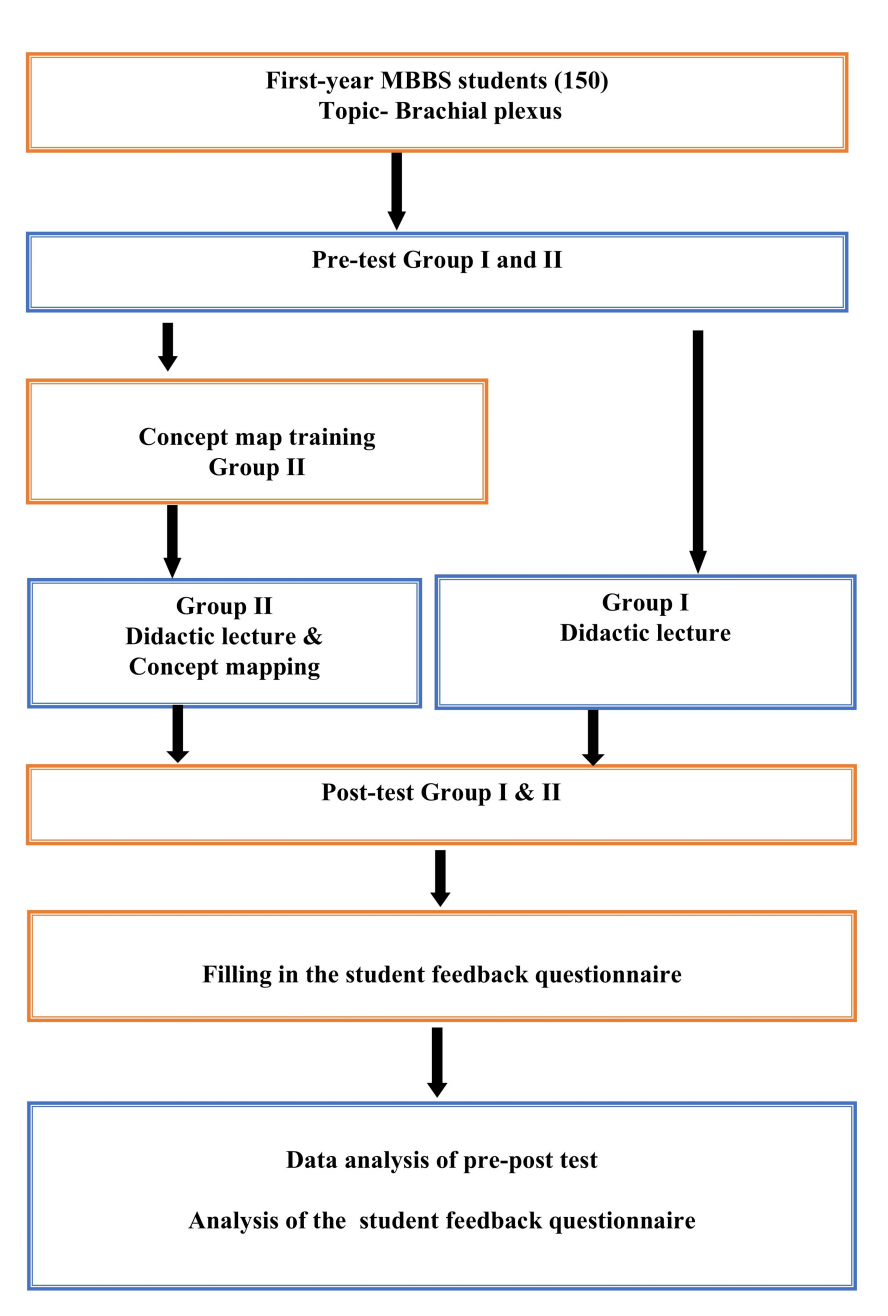
Study design

This cross-sectional, prospective study was conducted on 150 first-year MBBS medical students in the Department of Anatomy at MGUMST, Jaipur, during the 2024-2025 academic year, following ethical approval from the institution's Ethics Committee. One hundred fifty students were divided into two groups, group I (75 students) and group II (75 students), using Random Allocation software (Version 2.0, February 2025). Groups were assigned the topic of brachial plexus by using traditional didactic lectures and developing concept maps. A group of 75 students was taught using only traditional didactic lectures (Figure 1).

Pre- and posttests were conducted for all groups with a prevalidated questionnaire. Feedback was obtained from the students of each group on a three-point Likert scale. The study employed a combination of quantitative and qualitative assessments. 

Study design

Prestudy Phase

One hundred fifty students were divided into two equal groups: group I, didactic lecture; and group II, didactic lecture and concept mapping (Figure 1). The study team carefully made an assessment plan and feedback methods for evaluating learning outcomes. Before starting the workshop on CMs, a didactic lecture on the brachial plexus and its clinical anatomy was given for all 150 students (Figure 1). Topics included the brachial plexus and its clinical anatomy: formation, branches, motor and cutaneous nerve supply, and lesions.

Group II students were first given guidelines to make a CM, which included what a concept map is and how to understand a topic using a concept map, and some CMs related to the topic were discussed with them. The procedure to make a concept map and the design of CMs were also explained to them.

Session: The concept mapping session started with an initial briefing. Facilitators introduced students to the core principles and benefits of active learning. After this, they prepared concept maps to illustrate the very important and vast topic of the brachial plexus and its clinical anatomy. During the submission phase, students made the concept maps during two hours of exercise; after that, they submitted them to the Google Classroom platform, where they submitted their subject material into conceptual work.

Pre- and posttests were prepared and taken in both groups I and II. A structured feedback questionnaire was given to the students who were exposed to concept map making. It contained nine closed-ended questions. This feedback was made to assess learning through concept mapping as a teaching-learning method and to evaluate students’ perception of the teaching method. The Likert scale of three points was chosen for evaluating the CMs' feedback.

Statistical analysis

The data from pretests and posttests were tabulated in a spreadsheet program (MS Excel (Microsoft Corporation, Redmond, Washington, United States)). Statistical analysis was done using PAST® software version 4.03. Improvement in test scores in both teaching methods was also analyzed by deducting the pretest score from the posttest score for each student and tabulating the results in Microsoft Excel. The Shapiro-Wilk test was performed to assess the Gaussian fit of the variables. Values were expressed as mean ± SD or median [interquartile range {Q1(25%)-Q3(75%)}] depending on the normality of data. Paired t-tests and Wilcoxon matched-pairs signed-rank tests were applied for statistical analysis according to the distribution of data. A p-value of <0.05 was considered statistically significant.

## Results

A total of 150 students participated in the study. The students were divided into group I and group II.

Comparison of pretest and posttest in group I

**Figure 2:**
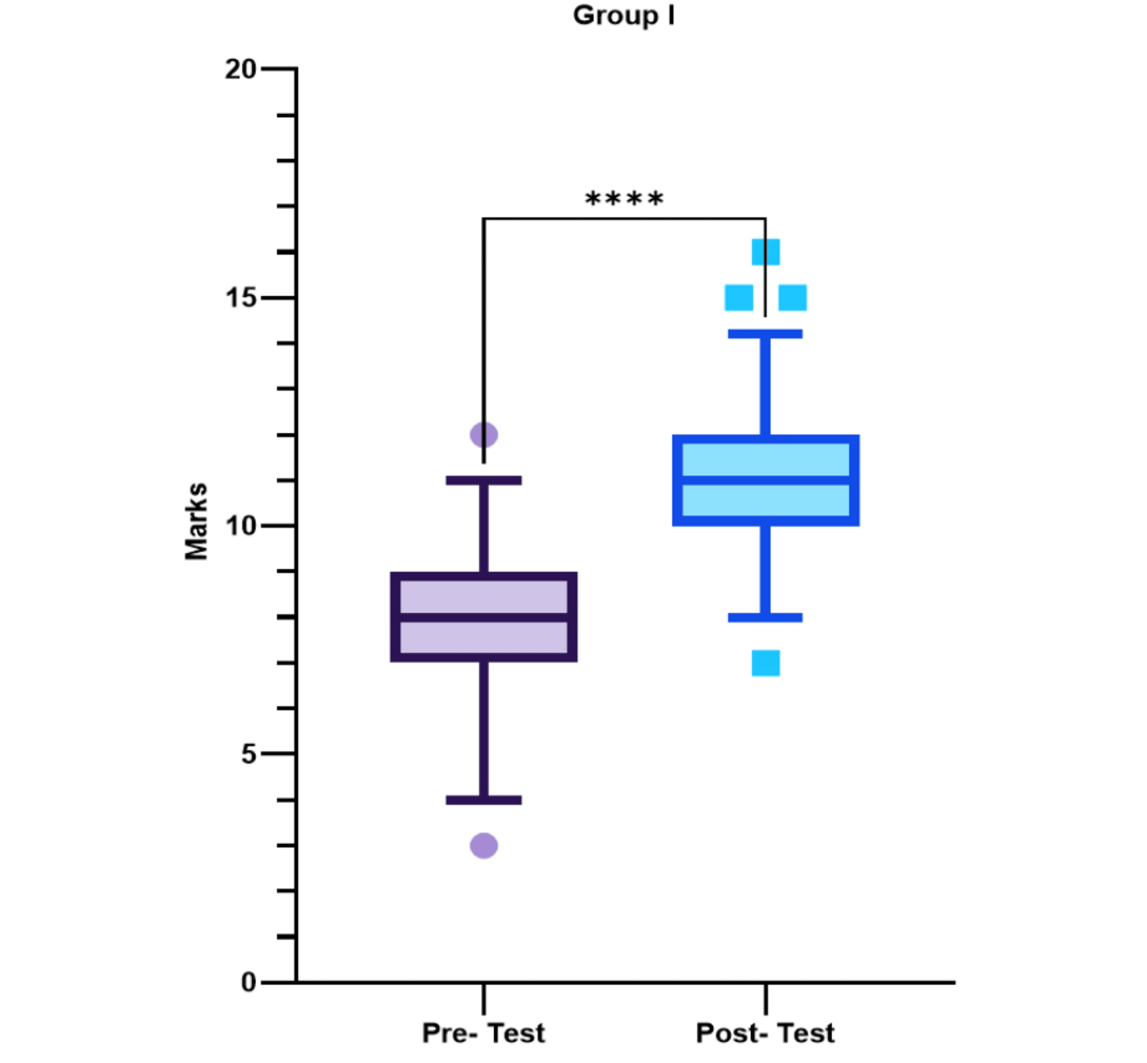
Comparison of pretest and posttest scores of group I depicted as median (Q1-Q3). Whiskers represent 5th and 95th percentiles, respectively. Symbols outside whiskers represent outliers. ****very highly significant

A Wilcoxon matched-pairs signed-rank test was performed to compare the pretest and posttest marks in group I. In group I, the median (interquartile range) marks of the pretest in 75 students were found to be 8 (7.0-9.0), whereas in the posttest, it was 11 (7.0-12.0) (Figure 2, Table 1). The increase in the student performance was found to be very highly significant in group I after the lecture (p-value < 0.0001) (Figure 2) (Table 1).

**Table 1:** Pretest and posttest scores in group I and group II

Group	Pretest marks (median (25%-75%))	Posttest marks (median (25%-75%))	p- value
Group I	8 (7.0-9.0)	11 (7.0-12.0)	<0.0001
Group II	8 (7.0-9.0)	15 (14.0-16.0)	<0.0001

Comparison of pretest and posttest in group II

**Figure 3:**
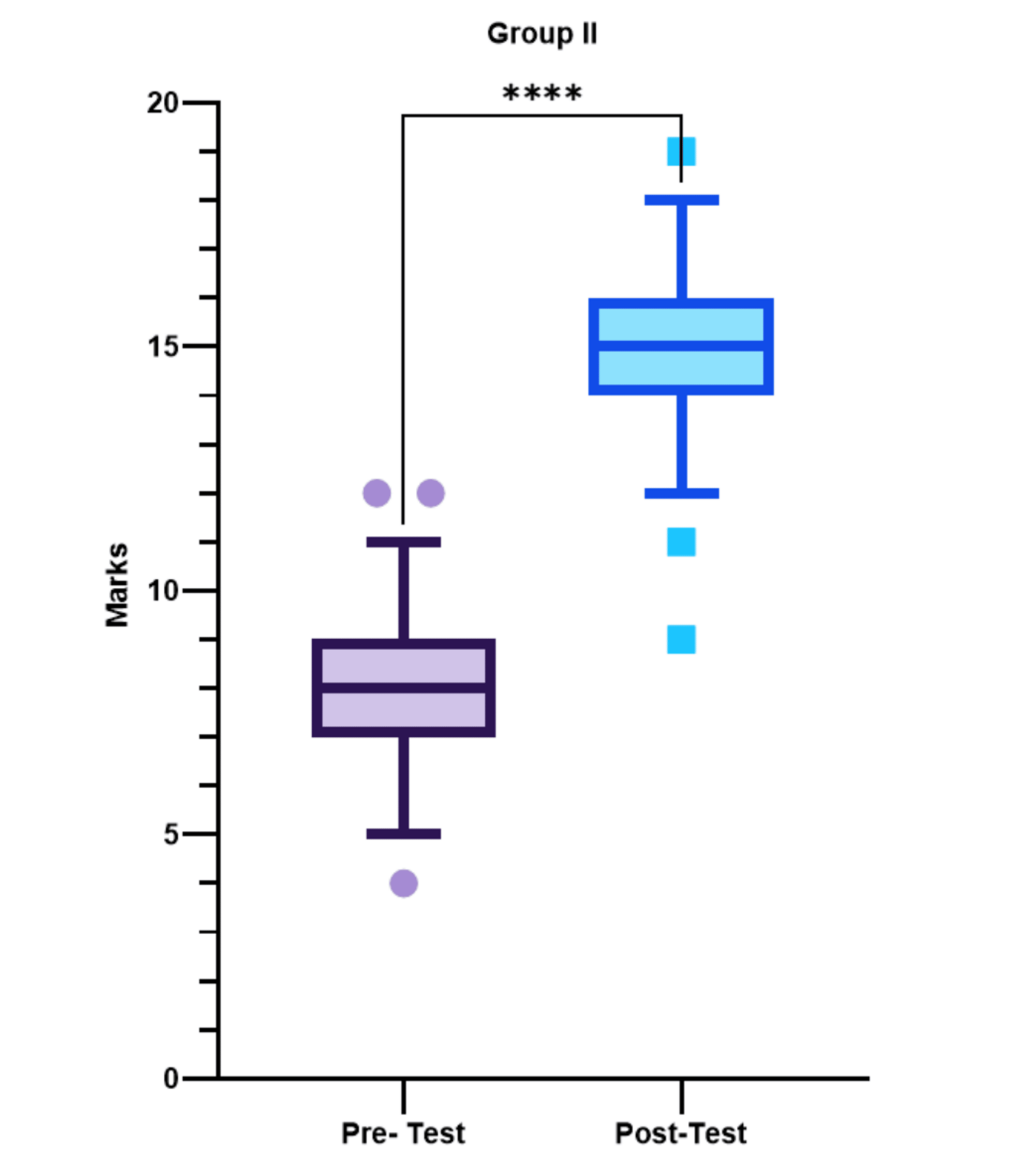
Comparison of pretest and posttest scores of group II depicted as median (Q1-Q3). Whiskers represent 5th and 95th percentiles, respectively. Symbols outside whiskers represent outliers ****very highly significant

A Wilcoxon matched-pairs signed-rank test was performed to compare the pretest and posttest marks in group II. In group II, the median (interquartile range) marks of the pretest in 75 students were found to be 8 (7.0-9.0), whereas in the posttest, it was 15 (14.0-16.0) (Figure 3, Table 1). The increase in the student performance was found to be highly significant in group I after the lecture (p-value < 0.0001) (Figure 3, Table 1). 

Comparison of the improvement of performance of students (posttest-pretest marks) between group I and group II

**Figure 4:**
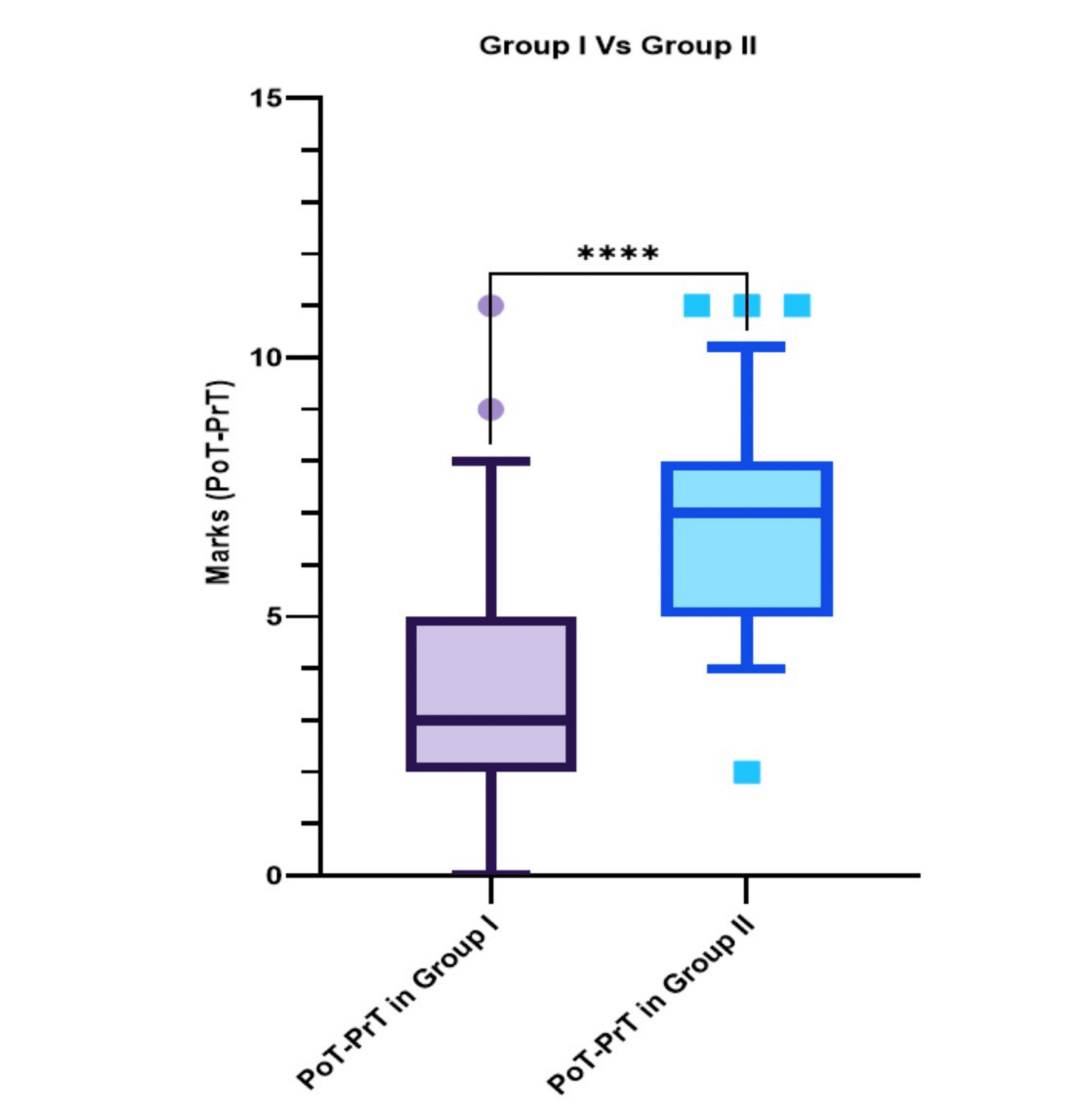
Comparison of improvement of marks (pretest-posttest scores) between group I and group II depicted as median (Q1-Q3). Whiskers represent 5th and 95th percentiles, respectively. Symbols outside whiskers represent outliers PoT: posttest marks; PrT: pretest marks; ****very highly significant

A Mann-Whitney test was performed to compare the improvement of performance in terms of gain of marks (posttest-pretest marks of students) between group I and group II. We observed a significant increase in the performance of group II students (median = 7 (5.0-8.0)) in comparison to group I students (median = 3 (2.0-5.0)) (p-value < 0.0001) (Figure 4).

**Figure 5:**
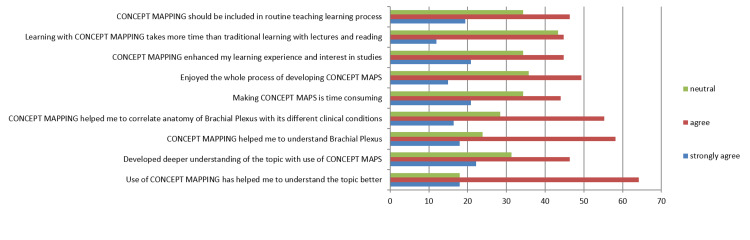
Analysis of feedback questionnaire showing percentage of student accepting concept mapping aid in enhancing analytical thinking

Data shows 46.3% (70) of students agreed that a newer method should be included in the regular study of medical sciences. A total of 49.3% (74) and 44.8% (67) of students agreed that they enjoyed the process of developing a CM and that it enhanced their learning experience, respectively. Though it takes some more time to complete the exercise. 64.2% (96) and 55.2% (83) agreed that this exercise helps you to understand and correlate the topic more coherently, respectively. A total of 46.3% (70) agreed that it is necessary for deeper learning of the topic (Figure 5).

## Discussion

In this study, we observed that group II students obtained 20% more marks in comparison to group I. The findings of our study helped us to understand that giving a CM to students eases their learning as well as their deeper understanding and memorization of the topic. The visual presentation of the brachial plexuses eases students into long-term learning and memorization of the topic. CMs were used in the medical curriculum long ago and helped in raising the learning levels [7]. Mere learning of a topic is easily forgotten, so it should not be rote learning but rather meaningful learning, as stated by David Ausubel’s assimilation theory [3]. The study conducted by Hung and Lin demonstrated that the introduction of an integrated CM helps students in problem-based learning (PBL) [8]. Novak et al. used the CM with the technology Concept Map tool. CMs help in building the three domains of learning, turning the learning into advantageous learning for students [3]. Narang et al. found that CMs aid in identifying the problem, learning important concepts, longer-term learning, and engagement of students [1]. Another study done by Fonseca et al. also used the CM tool for developing a CM template for pathophysiology mechanisms between clinical cases used by medical students [7].

Cross-links in CMs aid in raising student knowledge from one domain to another. It thereby leads to a higher degree of learning with better cognitive performance. This study established that the medical curriculum should adopt active learning methods to make students lifelong learners. This was concluded by Beigzadeh et al. [9]. Chand et al. did their study on 250 students divided into groups of 20-22. They did their evaluation using pre- and posttests with feedback. The result showed that introducing CMs led to a highly significant positive result [10]. Shanmugarajah et al. experimented with first-year MBBS students (n = 110), who demonstrated the feasibility of using CMs. How do the CMs help them? They integrated the knowledge with understanding, leading to active learning and critical thinking. This not only helped them to integrate knowledge but also helped in raising the scores of students in the assessment. It was found to be more liked by students, as it has raised student engagement and better understanding than traditional learning [11]. Another study was conducted on medical students, divided into two groups: a control group and a CM group. The outcome was tested by taking a surprise test. The result was better output or marks in the interventional group, concluding that CMs are a better learning tool [12].

A study done involving 128 medical students by Srikantaiah et al. also reflected the CM as a good pedagogical tool to enhance learning [2]. A systematic review carried out using eight databases also reflected the better integration of knowledge and outcome based on CM formation, which leads to better critical thinking in undergraduate medical students. Concept mapping is considered a better teaching and learning tool in medical education. A study by Sargolzaie et al. on 80 medical interns, where one group was trained by concept mapping and the other by book reading, showed a significant mean difference in the CM group [13]. Baliga et al. did a study on third-year medical students. This was done in two stages: first, a pretest was taken to assess their knowledge. After that lecture, learning through a CM was done. A later post-test was done. There was a significant difference between the two groups, with CM students having better meaningful learning [14]. Loizou et al. gave first-year medical students questions to solve without any prior knowledge of CM making. They were given PBL to prepare CMs. Later, they wrote semi-structured critical and clinical thinking questionnaires. CMs led to more critical thinking and gained more in-depth knowledge. The result of the questionnaires provided reflects that by using CMs, the students recall memory, organize material concisely, are well-prepared for their PBL sessions, and serve as a valuable revision resource [6].

CMs serve as a versatile tool across medical education, supporting both undergraduate and postgraduate learning. They begin with topic selection, followed by idea generation, organization, and ultimately the integration of concepts to form a coherent and insightful structure [11]. These maps help bridge the gap between traditional classroom instruction and a deeper understanding of content. The comparison of pre- and posttest results in the present study highlights how concept mapping aids in aligning foundational science knowledge with clinical case interpretation. This approach enhances scenario-based learning and demonstrates its broad educational value.

Limitations of the study

In this study, the advantage of concept making was assessed in a small sample size and only on a small topic among the same institute students. In the future, this study should be planned on a large sample size among different institute students.

## Conclusions

As a helpful tool for student learning, concept mapping may be included in the teaching and learning process. It also connects vertically with clinical sciences and aids in the correlation of the foundational MBBS courses. Students' creativity is enhanced by concept mapping, which increases their ability to analyze situations cognitively. Concept maps are beneficial to both students and teachers because they enable the identification of areas in which students lack comprehension and give instructors a model of how students think, so they may give insightful comments to guide study.

## Figures and Tables

**Figure 6:**
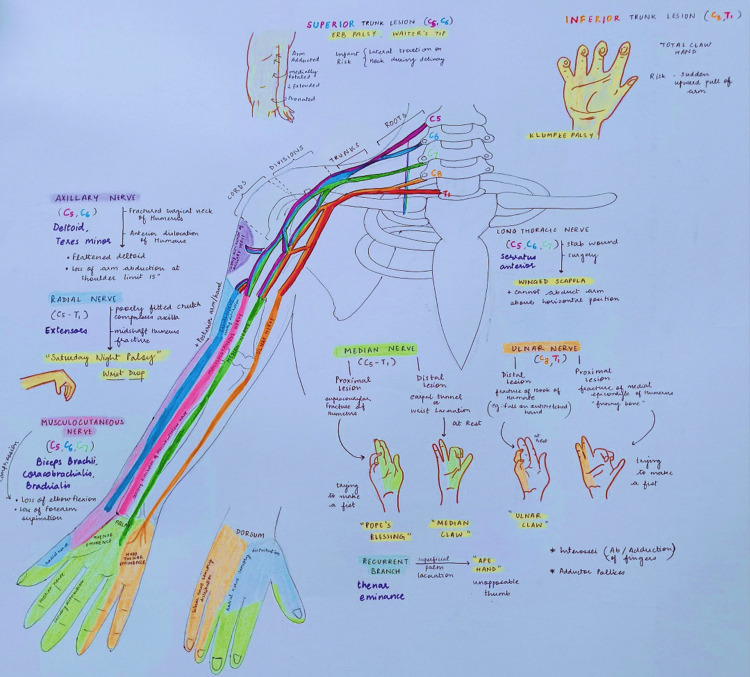
Concept map drawn by student on topic of brachial plexus formation and its clinical anatomy (image credits: Neharika)

## Appendices

The students prepared concept maps to illustrate the brachial plexus and its clinical anatomy under the guidance of facilitators (Figure 6).

## References

[1] Narang KK, Lata P (2024). The construction of concept maps: enhancing learning and knowledge representation. ShodhKosh J Vis Per Arts.

[2] Chikkarahalli SV, Mathada VR, Gowdappa DV (2025). Enhancing anatomy learning: A concept map-based approach for first-term MBBS students. Educ Med.

[3] Novak JD, Gowin DB (1984). Learning How to Learn.

[4] Michael J (2001). The Claude Bernard distinguished lecture: in pursuit of meaningful learning. Adv Physiol Educ.

[5] Edmondson KM (1995). Concept mapping for the development of medical curricula. J Res Sci Teaching.

[6] Loizou S, Nicolaou N, Pincus BA, Papageorgiou A, McCrorie P (202212). Concept maps as a novel assessment tool in medical education. MedEdPublish.

[7] Fonseca M, Oliveira B, Carreiro-Martins P, Neuparth N, Rendas A (2020). A concept map template to be used by medical students for displaying pathophysiological mechanisms within clinical cases. MedEdPublish (2016).

[8] Hung CH, Lin CY (2015). Using concept mapping to evaluate knowledge structure in problem-based learning. BMC Med Educ.

[9] Beigzadeh A, Haghani F (2015). Concept maps and meaningful learning in medical education. Stri Develop Med Educ.

[10] Chand L, Sowmya K, Silambanan S, Manikandan Manikandan (201853135). Meaningful learning in medical science by self-directed approach of-concept mapping. J Educ Technol Health Sci.

[11] Shanmugarajah MM, Mondal H, Das T (2024). From fragmented facts to unified knowledge: exploring concept mapping in neuromuscular physiology among first-year medical students. Cureus.

[12] Sorte SR, Sande S, Rathod SB, Vij VA, Gumashta J, Muthiyan G, Patil A (2024). Concept mapping a potential pedagogical strategy to foster meaningful learning in physiology students. J Educ Health Promot.

[13] Sargolzaie N, Sargazi S, Lotfi G (2019). Concept mapping as a tool to improve medical student's learning about rabies surveillance. J Educ Health Promot.

[14] Baliga SS, Walvekar PR, Mahantshetti GJ (2021). Concept map as a teaching and learning tool for medical students. J Educ Health Promot.

